# An *N*-Terminal Fragment of Yeast Ribosomal Protein L3 Inhibits the Cytotoxicity of Pokeweed Antiviral Protein in *Saccharomyces cerevisiae*

**DOI:** 10.3390/toxins6041349

**Published:** 2014-04-11

**Authors:** Rong Di, Nilgun E. Tumer

**Affiliations:** Department of Plant Biology and Pathology, School of Environmental and Biological Sciences, Rutgers University, New Brunswick, NJ 08901, USA; E-Mail: tumer@aesop.rutgesr.edu

**Keywords:** pokeweed antiviral protein, ribosomal protein L3, ribosome inactivating protein, α-sarcin/ricin loop

## Abstract

We have previously shown that ribosomal protein L3 is required for pokeweed antiviral protein (PAP), a type I ribosome inactivating protein, to bind to ribosomes and depurinate the α-sarcin/ricin loop (SRL) in yeast. Co-expression of the *N*-terminal 99 amino acids of yeast L3 (L3Δ99) with PAP in transgenic tobacco plants completely abolished the toxicity of PAP. In this study, we investigated the interaction between PAP and L3Δ99 in *Saccharomyces cerevisiae*. Yeast cells co-transformed with PAP and L3Δ99 showed markedly reduced growth inhibition and reduced rRNA depurination by PAP, compared to cells transformed with PAP alone. Co-transformation of yeast with PAP and L3Δ21 corresponding to the highly conserved *N*-terminal 21 amino acids of L3Δ99, reduced the cytotoxicity of PAP. PAP mRNA and protein levels were elevated and L3Δ99 or L3Δ21 mRNA and protein levels were reduced in yeast co-transformed with PAP and L3Δ99 or with PAP and L3Δ21, respectively. PAP interacted with L3Δ21 in yeast cells *in vivo* and by Biacore analysis *in vitro*, suggesting that the interaction between L3Δ21 and PAP may inhibit PAP-mediated depurination of the SRL, leading to a reduction in the cytotoxicity of PAP.

## 1. Introduction

Pokeweed antiviral protein (PAP), isolated from the leaves of pokeweed plants (*Phytolacca americana*), is a type I ribosome inactivating protein (RIP). PAP removes a specific adenine from the highly conserved, α-sarcin/ricin loop (SRL) in the large rRNA [1,2], a process termed depurination. Depurination of the SRL inhibits the eEF-2 (elongation factor 2)-catalyzed GTP (guanosine-5'-triphosphate) hydrolysis and translocation of the peptidyl-tRNA to the P-site during protein synthesis [3,4]. We have previously shown that PAP binds to ribosomal protein L3 (RPL3) in order to gain access to the SRL to depurinate the 25S rRNA in yeast [5]. L3 is a highly conserved protein associated with the peptidyltransferase center of ribosomes. We showed that ribosomes of yeast cells harboring the *mak8-1* allele of L3 are resistant to depurination by PAP [5]. Co-expression of the full-length yeast L3 with wild type (wt) PAP in transgenic tobacco plants reduced the cytotoxicity of PAP, while co-expression of the *N*-terminal 99 amino acids of yeast L3 (L3Δ99) with wt PAP abolished the cytotoxicity of PAP in transgenic tobacco plants [6]. PAP can inhibit translation of capped mRNAs and viral RNAs by recognizing the cap structure and depurinating the capped RNAs [2], suggesting that the SRL is not the only substrate of PAP. In this study, we show that co-expression of yeast L3Δ99 with PAP in *Saccharomyces cerevisiae* reduces the cytotoxicity of PAP. We further demonstrate that the highly conserved *N*-terminal 21 amino acids of L3Δ99 (L3Δ21) are able to reduce the cytotoxicity of PAP. PAP binds to L3Δ21 in yeast cells *in vivo* and by Biacore analysis *in vitro*, suggesting that this interaction may block ribosome depurination by PAP in the cytosol, leading to a reduction in the cytotoxicity of PAP.

## 2. Materials and Methods

### 2.1. Plasmids and Yeast Growth Conditions

PAP and the active site mutant PAP_E167V_ expression plasmids were described previously [5]. The cDNA encoding the *N*-terminal 99 amino acids of yeast L3 (L3Δ99) was cloned into pAC55 with *URA3* marker. PAP, PAP_E167V_ and L3Δ99 are under control of the galactose-inducible *GAL1* promoter. L3Δ99 cDNA was cloned into pYES2.1HisV5 (Life Technologies, Grand Island, NY, USA) with *URA3* marker under the *GAL1* promoter, resulting in L3Δ99V5 plasmid. The cDNA encoding the *N*-terminal 21 amino acids of yeast L3 was cloned into pYES2.1HisV5, resulting in L3Δ21V5 plasmid. These plasmids were transformed into *S. cerevisiae* strain W303 (*MATa ade2-1 trp1-1 ura3-1 leu2-3*, *112 his3-11*, *15 can1-100*). Transformed yeast cells were grown in the synthetic dropout (SD) medium (0.67% Bacto-yeast nitrogen base) supplemented with the appropriate amino acids. To induce the expression of PAP and L3Δ, yeast cells were initially grown at 30 °C in selective medium containing 2% glucose until *A*_600_ of 0.6. The cells were then pelleted and re-grown in the selective medium containing 2% galactose at 30 °C.

### 2.2. Yeast Cell Viability Assay

Yeast cells were sampled at 0, 4, 6, and 10 h post induction with galactose. A 10-fold serial dilution of cells was plated onto selective medium containing 2% glucose using 15 μL of cells at 0.1 *A*_600_ unit per mL as the starting concentration. Cells were photographed after 72 h of growth at 30 °C.

### 2.3. Cell Fractionation and Immunoblot Analysis of Protein Expression

Total protein was extracted from yeast as described [5]. Yeast cells were spheroplasted and fractionated according to Kornizer [7] to isolate the membrane and cytosolic fractions. Equal amount of protein samples (15 μg each) were separated by a 12% SDS-PAGE, transferred to nitrocellulose membrane and probed with anti-PAP polyclonal antibody to detect the expression of PAP. Chemiluminescence (PerkinElmer Life Sciences, Walham, MA, USA) was used to visualize the protein expression. The blots were stripped for 30 min with 8 M guanidine hydrochloride and re-probed with anti-V5 monoclonal antibody (Life Technologies, Grand Island, NY, USA) for the expression of L3Δ99 or L3Δ21, or with anti-G6PD (glucose-6-phosphate dehydrogenase) monoclonal antibody (Life Technologies, Grand Island, NY, USA) for the cytosolic protein expression. Additionally, anti-Dpm1p (dolichol phosphate mannose synthase) monoclonal antibody (Life Technologies, Grand Island, NY, USA) was used for the membrane protein expression and anti-Pgk1p (3-phosphoglycerate kinase) monoclonal antibody (Life Technologies, Grand Island, NY, USA) was used for the cytosolic protein expression.

### 2.4. rRNA Depurination Assay

Ribosomal RNA depurination was assayed by a dual primer extension assay as previously described [8]. ^32^P-ATP-labeled extension products were separated on a 7 M urea, 5% polyacrylamide gel and quantified using a Phosphorimager (GE Healthcare, Little Chalfont, Buckinghamshire, UK).

### 2.5. Real-Time PCR Analysis

Total yeast RNA was isolated by the hot-phenol method as described [8]. One microgram of total RNA was used to synthesize cDNA with random primer and SuperScript II reverse transcriptase (Life Technologies, Grand Island, NY, USA). Relative real-time PCR assay was performed with SYBR green master mix and the ABI 7000 Sequence Detection System (Applied Biosystems/Life Technologies, Grand Island, NY, USA) using the following gene-specific primers: 5’-GGGTAAGATTTCAACAGCAATTCA-3’ (forward primer) and 5’-CACCACTGGCATCC-ACTAGCT-3’ (reverse primer) for PAP, 5’-GGTTACGTCGAAACCCCAAGAGG-3’ (forward primer at the end of L3Δ99) and 5’-AGAGGGTTAGGGATAGGCTTACCTT-3’ (reverse primer at the V5 tag of pYES2.1 vector) for L3Δ99V5, 5’-GGTTTCTTGCCAAGAAAGAGAAAG-3’ (forward primer at the end of L3Δ21) and 5’-AGAGGGTTAGGGATAGGCTTACCTT-3’ (reverse primer at the V5 tag of pYES2.1 vector) for L3Δ21V5, 5’-CAGCAATGACTTTCAACATCGAA-3’ (forward primer) and 5’-CCGGCACGCATCATGAT-3’ (reverse primer) for G6PD as internal control. Another set of primers were used for the yeast actin gene: 5’-CACCAACTGGGACGATATGGA-3’ (forward primer) and 5’-GGCGACTCTCAATTCGTTGTAGA-3’ (reverse primer).

### 2.6. Purification of PAP Protein from PAP or PAP/L3*Δ99* Yeast Cells

PAP protein was isolated from yeast cells transformed with PAP and PAP/L3Δ99 as described [9]. Briefly, yeast cells were broken by glass beads. Cell extracts were clarified by Sephadex G-25 superfine gel (GE Healthcare, Little Chalfont, Buckinghamshire, UK) column in WCE buffer (30 mM HEPES, pH 7.4, 100 mM potassium acetate, 2 mM magnesium acetate and 2 mM dithiothreitol) supplemented with 0.5 mM PMSF. Subsequently the clarified cell extracts were concentrated with Microcon YM-10 columns (Millipore, Billerica, MA, USA).

### 2.7. Surface Plasmon Resonance (SPR) Analysis

A BIAcore 3000 SPR-based biosensor system (Biacore/GE Healthcare, Little Chalfont, Buckinghamshire, UK) was used to measure the kinetics of the interaction between PAP as the analyte and the yeast L3Δ21 peptide as the ligand. The yeast L3Δ21 peptide was synthesized by IDT Inc. (Coralville, IA, USA) with *N*-terminal biotinylation. A protocol described by Rajamohan [10] was followed with modifications. For streptavidin-biotin coupling, 30 μL biotinylated L3Δ21 peptide at 4 μg/μL was injected at a flow rate of 5 μL/min in HBS-EP buffer (0.1 M HEPES, pH 7.4, 0.15 M NaCl, 3 mM EDTA, and 0.005% polysorbate 20) and coupled onto Fc2 of a Biacore SA sensor chip. Biotin in HBS-EP buffer (25 μg/mL, 120 μL total) was injected over Fc1 to block the unoccupied surfaces at a flow rate of 5 μL/min. His-tagged PAP purified from *E. coli* BL21(DE3)-pLysS cells was used in this study [9]. Different concentrations of PAP (90 μL) prepared in HBS-EP buffer were injected at a flow rate of 30 μL/min onto Fc1 and Fc2, followed by a dissociation time of 5 min. This kinetic study was carried out at 25 °C with HBS-EP as the running buffer. The binding surfaces were regenerated between samples by injection of 50 μL of 1 M NaCl at a flow rate of 50 μL/min and washed by 300 μL HBS-EP at a flow rate of 100 μL/min. To analyze data, base lines for all curves were adjusted to zero, and the injection times were aligned. Specific binding curves were produced by subtracting the Fc1 background sensorgrams from the Fc2 experimental sensorgrams. The association and the dissociation phases of the aligned binding curves were fit simultaneously, assuming a bimolecular reaction between soluble PAP and the immobilized biotinylated L3Δ21, equivalent to the Langmuir isotherm for absorption to a surface. The fit was assessed by the low statistical χ^2^ value and the residues that were randomly distributed around zero. The BIAevaluation software (version 4.0, Biacore/GE Healthcare, Little Chalfont, Buckinghamshire, UK) was used to calculate the association and dissociation rate constants by nonlinear fitting of the primary sensorgram data. The binding affinities of PAP to L3Δ21 were calculated from the rate constants and from analyzing the equilibrium binding.

### 2.8. Co-Immunoprecipitation

The membrane and cytosolic fractions of yeast cells expressing PAP and PAP/L3Δ99V5 were used as substrates for co-immunoprecipitation with the polyclonal antibody against PAP based on protocols previously described [5,11]. The complexes of antibody and membrane or cytosolic fractions were incubated with protein A-Sepharose beads (GE Healthcare, Little Chalfont, Buckinghamshire, UK) which were subsequently washed with HEPES buffer containing 0.25 M NaCl and 1% Triton 100. The complexes were then eluted with SDS sample buffer, electrophoresed on SDS-PAGE gel and visualized by immunoblot analysis with V5 monoclonal antibody (Life Technologies, Grand Island, NY, USA) and PAP polyclonal antibody.

## 3. Results

### 3.1. Cytotoxicity of PAP is Reduced in Yeast Cells Co-Transformed with PAP and L3Δ99

**Figure 1:**
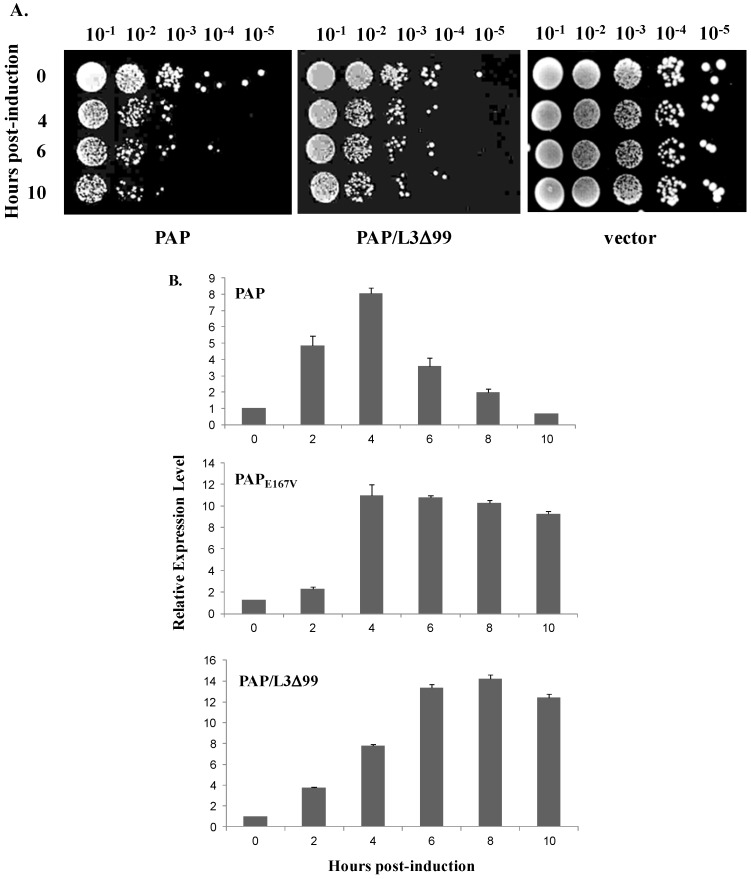
(**A**) Viability analysis of yeast expressing pokeweed antiviral protein (PAP), PAP/L3Δ99 and harboring the empty vector. Yeast was induced in liquid synthetic dropout (SD) medium containing galactose for the hours indicated, and serial dilutions were plated on non-inducing SD plates containing glucose; (**B**) Real-time PCR analysis in yeast cells expressing PAP, the active site mutant PAP_E167V_ and PAP/L3Δ99 for 10 h induction using PAP-specific primers. Total RNA was isolated after induction with galactose at the hours indicated. The expression level was normalized to yeast G6PD mRNA as an internal control.

PAP was placed under the regulation of the *GAL1* promoter and transformed into *S. cerevisiae* strain W303. As shown by the viability assay in Figure 1A, very few cells expressing PAP were viable at 10 hpi. However, PAP cytotoxicity was reduced in cells co-expressing PAP and L3Δ99 (PAP/L3Δ99) (Figure 1A). This result is consistent with our previous data which showed that co-expression of yeast L3Δ99 with PAP completely abolished the cytotoxicity of PAP in transgenic tobacco plants [6].

We have previously shown that PAP mRNA levels decrease as PAP protein accumulates in yeast [8]. By RNase protection assay, we discovered that by 10 h post-induction (hpi), PAP mRNA levels decreased to approximately 10% of the levels observed at 4 hpi. However, the active site mutant PAP_E167V_ mRNA levels increased up to 10 hpi and reached steady state levels after 10 h [8]. In this study, we used real-time PCR analysis to examine the expression level of PAP over a 10 h time course in yeast expressing PAP, PAP_E167V_ and PAP/L3Δ99 using G6PD as the internal control after demonstrating that G6PD levels did not change during the 10 h time course. The relative level of PAP mRNA in cells transformed with PAP increased to the highest level at 4 hpi and then decreased to the lowest level at 10 hpi (Figure 1B). The relative level of PAP_E167V_ mRNA also rose to the highest level at 4 hpi, and remained relatively steady after 4 h (Figure 1B). Figure 1B also shows that the expression pattern of PAP over 10 h in yeast co-transformed with PAP/L3Δ99 was similar to the expression pattern of the non-toxic mutant, PAP_E167V_. These results demonstrated that toxicity of PAP was reduced, and PAP mRNA accumulated to a greater level in yeast co-transformed with PAP/L3Δ99 compared to yeast transformed with PAP alone.

### 3.2. PAP Protein Expression Level is Elevated, but Ribosome Depurination is Reduced in Yeast Co-Expressing PAP and L3Δ99

**Figure 2:**
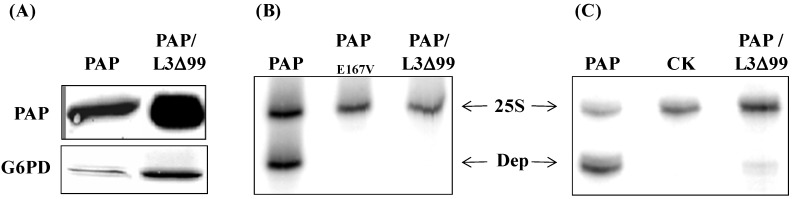
Analysis of PAP expression and ribosome depurination in yeast cells co-transformed with PAP and L3Δ99. (**A**) Immunoblot analysis of PAP expression. Cells were induced for 6 h with galactose. Total cell extracts (15 μg) were electrophoresed through 12% SDS-PAGE. Proteins were transferred to nitrocellulose membrane and probed with PAP polyclonal antibody (1:2000). The membrane was subsequently stripped and re-probed with G6PD monoclonal antibody (1:5000) as a loading control; (**B**) Analysis of ribosome depurination in yeast expressing PAP, PAP_E167V_ and PAP/L3Δ99 by primer extension analysis. The depurination primer (Dep) was used to measure the extent of depurination and the 25S primer (25S) was used to measure the amount of 25S rRNA; (**C**) *In vitro* depurination analysis of yeast rRNA using PAP protein isolated from yeast cells co-transformed with PAP/L3Δ99, compared to purified PAP protein (PAP). CK is untreated rRNA.

Immunoblot analysis of total yeast lysate using PAP-specific antibody showed that PAP protein level was elevated in yeast co-transformed with PAP/L3Δ99 (Figure 2A) compared to cells expressing PAP alone. Dual primer extension analysis demonstrated that rRNA depurination was abolished in cells co-transformed with PAP/L3Δ99, as in cells transformed with the active site mutant PAP_E167V _(Figure 2B). When PAP protein was isolated from cells co-expressing PAP/L3Δ99 and used in an *in vitro* depurination assay, PAP depurinated yeast ribosomes at a much reduced level (Figure 2C).

### 3.3. L3Δ21 Attenuates the Cytotoxicity of PAP

**Figure 3:**
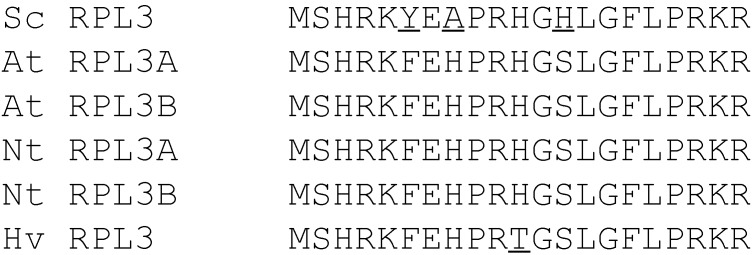
Alignment of the *N*-terminal 21 amino acids of L3 among different species. Sc = *Saccharomyces cerevisiae*, At = *Arabidopsis thaliana*, Nt = *Nicotiana tabacum*, Hs = *Homo sapiens*. Non-consensus amino acids are underlined.

**Figure 4:**
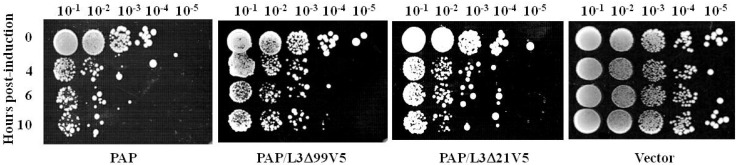
Viability analysis of yeast cells expressing PAP, PAP/L3Δ99V5, PAP/L3Δ21V5 or harboring the empty vector. Cells were induced in liquid SD medium containing galactose for the hours indicated, and serial dilutions were plated on non-inducing SD plates containing glucose.

The reduction in PAP cytotoxicity in yeast co-transformed with PAP/L3Δ99 prompted us to investigate the interaction between PAP and L3Δ99. To examine the level of L3Δ99 expression, L3Δ99 was cloned into pYES3.1/V5/His vector (Life Technologies, Grand Island, NY, USA) which contains the V5 epitope (L3Δ99V5). L3Δ99V5 was co-transformed into yeast with PAP (PAP/L3Δ99V5). Since the *N*-terminal 21 amino acids of yeast L3 are highly conserved among different species (Figure 3), we investigated if these amino acids were sufficient to reduce the cytotoxicity of PAP. L3Δ21V5, which contained the *N*-terminal 21 amino acids of yeast L3 with the V5 tag was co-transformed into yeast together with PAP. Viability analysis showed that L3Δ99V5 reduced the toxicity of PAP (Figure 4), although not as dramatically as L3Δ99 without the V5 tag (Figure 1A). L3Δ21V5 also reduced the cytotoxicity of PAP yeast co-transformed with PAP and L3Δ21V5 (Figure 4).

### 3.4. PAP mRNA and Protein Level is Elevated, L3Δ mRNA and Protein Level is Reduced in Yeast Co-Transformed with PAP and L3Δ

**Figure 5:**
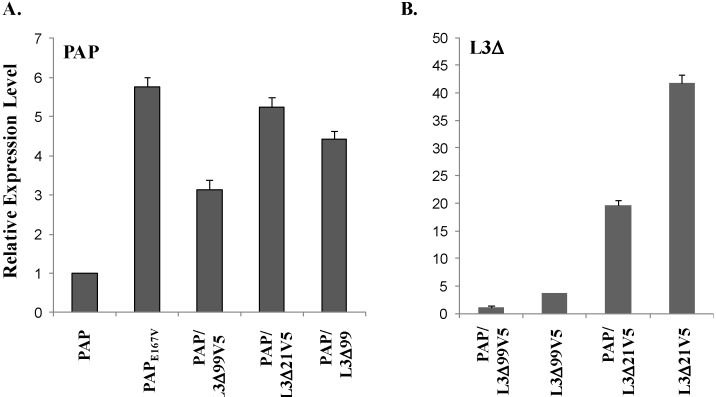
Real-time PCR analysis of PAP mRNA (**A**) and L3Δ99 or L3Δ21 mRNA (**B**) in yeast cells containing PAP, active site mutant PAP_E167V_, PAP/L3Δ99V5, PAP/L3Δ21V5 or PAP/L3Δ99. The expression level was normalized to yeast G6PD mRNA as an internal control. Total RNA was isolated after 6 h induction.

To determine if L3Δ reduces the cytotoxicity of PAP by affecting PAP expression, real-time PCR analysis was conducted using total RNA isolated from yeast cells singly or doubly transformed with PAP and L3Δ at 6 hpi. As shown in Figure 5A, the steady-state level of PAP mRNA in yeast cells transformed with the active site mutant PAP_E167V_ was 5-fold higher than that in cells transformed with wt PAP. PAP mRNA level in yeast co-transformed with PAP/L3Δ99V5 or PAP/L3Δ21V5 was approximately 2- and 4.5-fold higher, respectively than PAP mRNA level in yeast expressing PAP alone. We also examined the steady-state level of PAP mRNA in yeast co-transformed with PAP and L3Δ99 without the V5 tag. The PAP mRNA level in yeast co-transformed with PAP/L3Δ99 was approximately 3.5-fold higher than the level in cells expressing PAP alone. This indicates that when PAP was rendered non-toxic in yeast co-transformed with PAP/L3Δ99, PAP/L3Δ99V5 or PAP/L3Δ21V5, cells survived better and expressed elevated levels of PAP mRNA.

Real-time PCR analysis showed that the steady-state level of L3Δ99 mRNA in yeast transformed with L3Δ99V5 alone was 3-fold higher than in yeast co-transformed with PAP/L3Δ99V5 (Figure 5B). The L3Δ21 mRNA level in yeast transformed with L3Δ21V5 alone was approximately 20-fold higher than that in yeast co-transformed with PAP/L3Δ21V5. These data indicate that PAP mRNA levels increase and L3Δ mRNA levels decrease when yeast cells are co-transformed with PAP and L3Δ.

**Figure 6:**
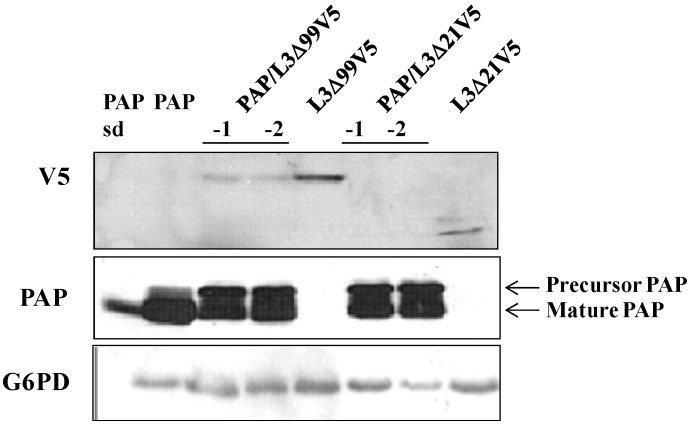
Immunoblot analysis of PAP and L3Δ protein in total extracts of yeast expressing PAP, PAP/L3Δ99V5, L3Δ99V5, PAP/L3Δ21V5 and L3Δ21V5. Total protein (15 μg) was separated on a 12% SDS-PAGE and transferred to nitrocellulose. The blot was initially probed with anti-V5 monoclonal antibody (1:5000) for the expression of L3Δ99V5 and L3Δ21V5 and then probed with PAP antibody (1:2000) after stripping. The membrane was then probed with G6PD monoclonal antibody (1:5000) for equal loading. Standard (sd.) denotes 100 ng of purified PAP from pokeweed plants (gift of Dr. Jim Irvin).

We have shown that PAP protein expression was elevated in in yeast co-transformed with PAP/L3Δ99 (Figure 2A), consistent with the elevated PAP mRNA level (Figure 5A) in these cells. Immunoblot analysis of total cell extracts 6 h post induction demonstrated that PAP protein expression was also elevated in yeast co-expressing PAP and L3Δ99V5 or L3Δ21V5 (Figure 6) compared to yeast expressing PAP alone. The mature form of PAP was detected in the total extract from yeast expressing PAP alone, while both the mature form and the precursor form were present in the total extracts from yeast co-expressing PAP and L3Δ99V5 or L3Δ21V5 (Figure 6). Additionally, Figure 6 shows that the L3Δ99V5 and L3Δ21V5 protein levels were reduced when they were co-transformed with PAP, compared to yeast transformed with only L3Δ99V5 or L3Δ21V5 (Figure 5B).

### 3.5. PAP Interacts with L3Δ Peptide

**Figure 7:**
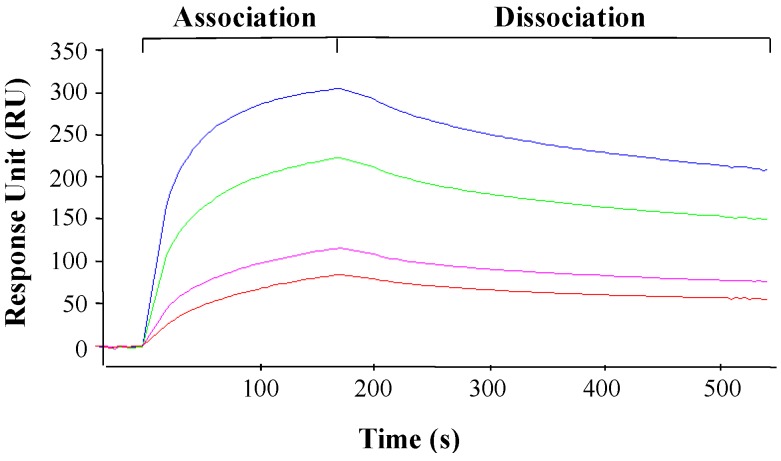
Biacore analysis of the interaction between PAP and biotinylated L3Δ21 peptide. Biotinylated L3Δ21peptide was immobilized onto the surface of a SA sensor chip. Recombinant PAP was passed over the flow cells at 103, 94, 75 and 45 pM using a Biacore 3000. The kinetic data was analyzed by the evaluation software (BIAevaluation version 4.0).

The alteration in PAP and L3Δ mRNA and proteins levels in yeast co-transformed with PAP and L3Δ suggested that PAP may interact with L3Δ. To determine if PAP interacts with L3Δ21 peptide, we used surface plasmon resonance (SPR) with a Biacore 3000. Biotinylated yeast L3Δ21 peptide was immobilized on a Biacore SA sensor chip and PAP was passed over the chip as the analyte. PAP bound to L3Δ21 in a 1:1 interaction model with a *k*_a_ of 1.86 × 10^3^ M^−1^S^−1^ and a *k*_d_ of 4.6 × 10^−5^ S^−1^ (Figure 7). The binding affinity (*K*_D_) of PAP for yeast L3Δ21 peptide was 24.8 nM.

**Figure 8:**
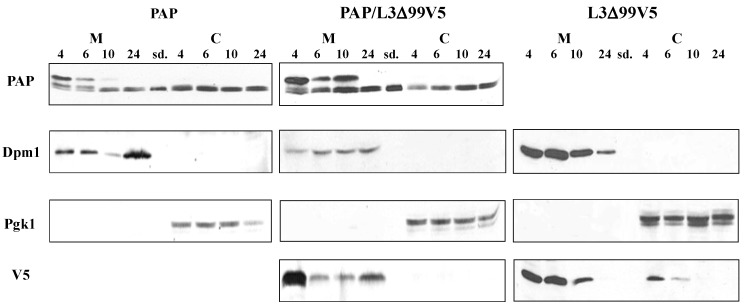
Immunoblot analysis of PAP and L3Δ99V5 protein in the membrane and cytosolic fractions of yeast cells expressing PAP, PAP/L3Δ99V5 and L3Δ99V5. Total protein (15 μg) was separated on a 12% SDS-PAGE and transferred to nitrocellulose. The blots were initially probed with PAP antibody (1:2000) or anti-V5 monoclonal antibody (1:5000). After stripping, the membranes were probed with anti-Dpm1 monoclonal antibody for membrane-specific protein expression, or with anti-Pgk1 monoclonal antibody for cytosolic-specific protein expression. Standard (sd.) denotes 100 ng of purified PAP from pokeweed plants (gift of Dr. Jim Irvin).

To determine if PAP interacted with L3Δ in yeast cells, we isolated the membrane and cytosolic fractions from yeast transformed with PAP or L3Δ99V5 alone, and from yeast co-transformed with PAP and L3Δ99V5 after induction for 4, 6, 10 and 24 h. Immunoblot analysis showed that both the precursor and the mature forms of PAP were present in the membrane fraction in yeast transformed with PAP at 4 h and 6 h post induction (Figure 8). The level of PAP precursor form in the membrane fraction was reduced at 10 h and diminished at 24 h post induction. Only the mature form of PAP was detected in the cytosolic fraction (Figure 8), consistent with previous observations [9]. In yeast co-transformed with PAP/L3Δ99V5, the precursor form of PAP was detected at a higher level in the membrane fraction at 10 h post induction compared to yeast expressing PAP alone (Figure 8). Our results also showed that L3Δ99V5 was present in both the membrane and cytosolic fractions in yeast expressing L3Δ99V5 alone. However, L3Δ99V5 was detected only in the ER membrane fraction in yeast co-transformed with PAP/L3Δ99V5 (Figure 8).

**Figure 9:**
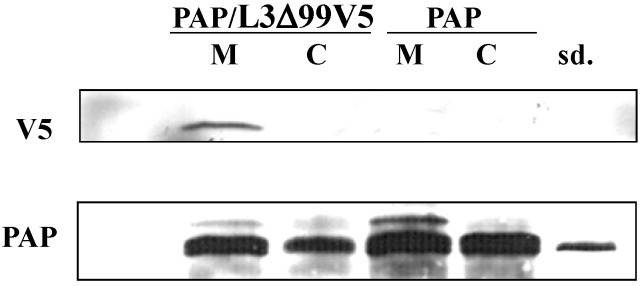
Co-immunoprecipitation of PAP with L3Δ99V5. Membrane and cytosolic fractions from yeast cells transformed with PAP and PAP/L3Δ99V5 were immunoprecipitated with PAP antibody at 6 h post induction and separated on a 12% SDS-PAGE, transferred to nitrocellulose and probed with anti-V5 monoclonal antibody (1:5000) and then re-probed with PAP antibody (1:2000). Standard (sd.) denotes 50 ng of purified PAP from pokeweed plants (gift of Dr. Jim Irvin).

To determine if PAP interacts with L3Δ99V5, we used co-immunoprecipitation analysis with the ER membrane and cytosolic fractions of yeast cells co-transformed with PAP/L3Δ99V5. As shown in Figure 9, PAP antibody could pull down L3Δ99V5 from the membrane fraction, but not from the cytosolic fraction in yeast co-transformed with PAP/L3Δ99V5, suggesting that PAP and L3Δ99 interacted in the membrane fraction.

## 4. Discussion

Although RIPs have the same specificity for the adenine in the SRL of the large rRNA subunit, they show different activity against ribosomes of different species. PAP is shown to be active equally on ribosomes from all five kingdoms, while ricin is much more active on rat liver ribosomes than on plant ribosomes [12]. Although the interaction between RIPs and ribosomal substrates has been stipulated to be the cause of these differences, the molecular recognition mechanism of RIPs for ribosomes was not well-understood until we showed that PAP gains access to ribosomes by binding to the ribosomal protein L3 [5]. Since L3 is highly conserved among different species, the interaction between PAP and L3 may account for the similar activity of PAP against ribosomes from different organisms [13]. We previously showed that co-expression of yeast L3Δ99 and PAP in transgenic tobacco plants completely abolished the cytotoxicity of PAP [6]. The rRNA was not depurinated in these transgenic plants. The mRNA level of PAP was up-regulated in PAP/L3Δ99 transgenic tobacco plants compared to transgenic plants expressing PAP alone. L3Δ99 mRNA was down-regulated in PAP/L3Δ99 transgenic tobacco plants compared to transgenic plants expressing L3Δ99 alone [6]. We envisioned two levels of interaction between PAP and L3Δ99 in the co-transformed tobacco plants. First, PAP may down-regulate L3Δ99 mRNA, instead of its own mRNA, resulting in a reduction in the L3Δ99 mRNA level and an increase in the PAP mRNA level. Second, the presence of L3Δ99 peptide may have contributed to the incorporation of suboptimal levels of L3 into ribosomes, resulting in a reduction in peptidyltransferase activity. Since actively translating ribosomes are the target of PAP, ribosomes with reduced peptidyltransferase activity might be less sensitive to PAP depurination.

In this study, we used the yeast model to further investigate the interaction between PAP and L3Δ99. We show that when PAP and L3Δ99 are co-expressed in yeast cells, the cytotoxicity of PAP is reduced (Figure 1A) and rRNA depurination is abolished (Figure 2B) even though PAP is expressed at a higher level in co-transformed yeast cells, than in yeast expressing PAP alone (Figure 2A). Real-time PCR analysis of yeast co-transformed with PAP/L3Δ99 indicated that PAP mRNA accumulated at the same level as the non-toxic active site mutant, PAP_E167V_ mRNA (Figure 1B). This may be because yeast cells expressing PAP/L3Δ99 or PAP_E167V_ are able to survive unlike yeast expressing PAP alone [14]. We show here that the conserved *N*-terminal 21 amino acids of yeast L3 are sufficient to reduce the cytotoxicity of PAP in co-transformed cells (Figure 4). The PAP mRNA level was higher in the PAP/L3Δ99V5 and PAP/L3Δ21V5 co-transformed cells compared to the cells transformed with only PAP, while the L3Δ mRNA level was lower in the co-transformed cells compared to cells transformed with only the L3Δ construct (Figure 5). The changes in the mRNA levels of PAP and L3Δ resulted in higher PAP protein levels (Figure 2A and Figure 6) and lower L3Δ99 and L3Δ21 protein levels, respectively (Figure 6). We have previously shown that PAP can target mRNA [15]. PAP binds to the m^7^GpppX cap structure and depurinates the mRNA downstream of the cap [15]. We have also demonstrated that PAP can inhibit translation of uncapped viral RNAs *in vitro* without causing detectable depurination of the viral RNA [16]. Our data suggest that PAP may target L3Δ99 and L3Δ21 mRNAs, instead of its own mRNA, resulting in a reduction in the level of these mRNAs and an increase in the level of PAP mRNA. We have previously shown that ribosome depurination is not sufficient for the cytotoxicity of PAP in yeast [17]. This may be why PAP cytotoxicity was partially reduced in yeast co-transformed with PAP/L3Δ99, although rRNA depurination was abolished (Figure 1A and Figure 2B).

Immunoblot analysis of the membrane and the cytosolic fractions of yeast cells transformed with PAP, L3Δ99V5 and PAP/L3Δ99V5 indicated that the subcellular distribution of PAP and L3Δ99V5 in the membrane and cytosol fractions was altered in co-transformed yeast cells (Figure 8). Both proteins were detected in the membrane fraction longer in co-transformed cells than in cells transformed with each protein alone. PAP interacted with L3Δ21 with high affinity (*K_D_* = 24.8 nM) as measured by SPR analysis (Figure 7). The co-immunoprecipitation results showed that PAP bound to L3Δ99V5 in the membrane fraction (Figure 9). We previously showed that both precursor and mature forms of PAP are associated with the membrane fraction in yeast [9]. We recently showed that the timing of the translocation of ricin from the ER to the cytosol is critical for its cytotoxicity [14]. The interaction between PAP and L3Δ99V5 on the membrane may interfere with the transport of PAP into the cytosol, leading to a reduction in rRNA depurination and cytotoxicity. Recent results indicate that PAP exists in the cytosol of pokeweed plant as a homodimer to allow pokeweed ribosomes to escape the depurination [17]. It is also possible that the formation of a PAP/L3Δ complex in co-transformed yeast cells reduces the enzymatic activity of PAP and compromizes its cytotoxicity.

We show here that co-expression of PAP with a 21 residue peptide corresponding to the first 21 amino acids of L3 inhibits depurination of the SRL and the cytotoxicity of PAP. Co-expression of PAP and L3Δ21 in yeast results in alteration of PAP and L3Δ21 mRNA and protein levels. PAP binds to L3Δ21 peptide, suggesting that the interaction between PAP and L3Δ21 prevents depurination of the SRL and reduces the cytotoxicity of PAP. In future studies mutagenesis can be carried out to identify the residues in L3Δ21 critical for its interaction with PAP.
